# Family‐based Association between Allele T of rs4646536 in *CYP27B1* and vitamin D deficiency

**DOI:** 10.1002/jcla.22898

**Published:** 2019-04-16

**Authors:** Songcheng Yu, Xing Li, Yan Wang, Zhenxing Mao, Yuanchen Xie, Lin Zhang, Chongjian Wang, Wenjie Li

**Affiliations:** ^1^ College of Public Health Zhengzhou University Zhengzhou China

**Keywords:** *CYP27B1*, family‐based association, rs4646536, transmission disequilibrium, vitamin D

## Abstract

**Background:**

The circulating concentration of 25(OH)D is widely applied to indicate the vitamin D status, as the directly metabolic genes of 25(OH)D, *CYP2R1,* and *CYP27B1* are associated with the concentration of 25(OH)D. However, the contributions of allelic transmission disequilibrium of single nucleotide polymorphisms (SNPs) in these genes to vitamin D deficiency remain unclear. We aimed at investigating the family‐based association between SNPs of *CYP2R1* and *CYP27B1* and vitamin D deficiency.

**Method:**

First, SNPs selected in family‐based study were screened by a pilot case‐control study. Second, allelic transmissions of the selected SNPs were investigated with family‐based study (n = 880). Finally, associations between selected SNPs and the concentration of 25(OH)D were verified in siblings (n = 120).

**Results:**

The results of the pilot case‐control study indicated that both CT and TT genotypes of rs4646536 in *CYP27B1* could increase the susceptibility of vitamin D deficiency when compared with CC genotype. The adjusted ORs were 2.846 (95%CI 1.312‐6.174, *P* = 0.008) and 2.609 (95%CI 1.197‐5.687, *P* = 0.016), respectively. Furthermore, the results of family‐based association test suggested that there was transmission disequilibrium for allele T of rs4646536 in vitamin D deficiency families. In addition, the concentration of 25(OH)D_3_ for CC genotype was higher than CT genotype between siblings (*P* = 0.016).

**Conclusions:**

Transmission disequilibrium of allele T of rs4646536 is associated with vitamin D deficiency.

**Highlights**

1. rs4646536 is associated with vitamin D deficiency in case‐control study.
2. Transmission disequilibrium for rs4646536 was found in vitamin D deficiency families.
3. Sibling with CT genotype of rs4646536 has lower levels of 25(OH)D_3_ than CC genotype.

## 1 INTRODUCTION

The circulating concentration of 25(OH)D is often applied to evaluate the vitamin D status. It is estimated that the heritability of 25(OH)D ranged from 23% to 80%.^1^ 25(OH)D is produced by hydroxylation at the C_25_ position of vitamin D in the liver, which is catalyzed by 25‐hydroxylase encoded by *CYP2R1*. 25(OH)D is further hydroxylated at the C_1_ position in the kidney to form 1,25(OH)_2_D, which is catalyzed by 1α‐hydroxylase encoded by *CYP27B1*,^2^ as the key metabolic genes of 25(OH)D, *CYP2R1,* and *CYP27B1* play direct roles in circulating concentration of 25(OH)D,^3^ which is often used as indicator of vitamin D status.^4^ Genetic variants in these genes have been reported to be associated with the concentration of circulating 25(OH)D. Bu et al reported that single nucleotide polymorphism (SNP) in *CYP2R1* was associated with 25(OH)D concentration in Caucasian.^5^ Significant associations between SNPs in *CYP27B1* and 25(OH)D concentration were observed by Orton.^6^ However, whether there are transmission disequilibriums for these genetic variants in vitamin D deficiency families remains unclear.

Transmission disequilibrium is used to describe the association between a genetic marker and a trait, which is tested by a family‐based study. In this study, we hypothesized that transmission disequilibrium of SNPs in *CYP2R1* and *CYP27B1* contributed to the heritability of 25(OH)D. Thus, we aimed at investigating the family‐based associations of SNPs in *CYP2R1* and *CYP27B1* with vitamin D deficiency. First, SNPs selected in family‐based study were screened by a pilot case‐control study. Second, allelic transmissions of the selected SNPs were investigated in a family‐based study. Finally, associations between selected SNPs having allelic transmission disequilibrium and the concentration of 25(OH)D were verified in siblings. This work would shed more light on the heritability of 25(OH)D.

## 2 MATERIALS AND METHODS

### 2.1 Study subjects

**Figure 1:**
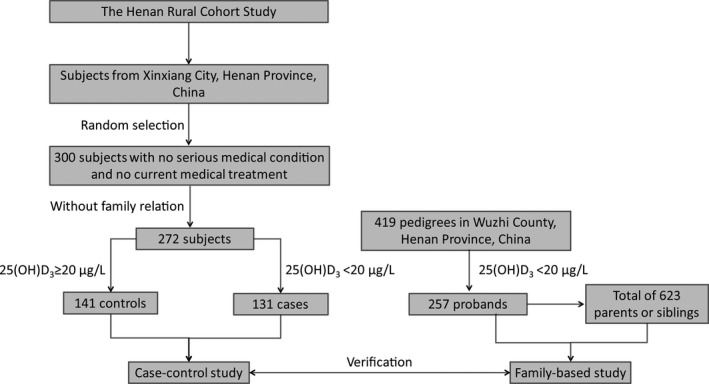
Flowchart of participant selection. A total of 272 subjects aged from 18 to 79 years were randomly selected from the Henan Rural Cohort Study for case‐control study. 257 pedigrees containing 880 subjects in Wuzhi County, Henan Province, China, were included in family‐based study

Participant flowchart is shown in Figure 1. A total of 272 subjects aged from 18 to 79 years were randomly selected from the Henan Rural Cohort Study for a pilot case‐control study, which has been registered at Chinese Clinical Trial Register (Registration number: ChiCTR‐OOC‐15006699). Detailed information is available at the website: http://www.chictr.org.cn/showproj.aspx?proj=11375.

A total of 257 pedigrees containing 880 subjects in Wuzhi County, Henan Province, China, were included in family‐based study. Their peripheral blood samples were collected for 25(OH)D_3_ measurement and DNA extraction. Concentration of 25(OH)D_3_ below 20 μg/L was defined as vitamin D deficiency.

The protocol was reviewed and approved by Life Science Ethics Review Committee of Zhengzhou University. All procedures performed in studies involving human participants were in accordance with the ethical standards of the institutional and/or national research committee and with the 1964 Helsinki declaration and its later amendments or comparable ethical standards. Informed consent was obtained from all individual participants.

### 2.2 25(OH)D_3_ measurement

A certificated third‐party medical laboratory of Kingmed Center for Clinical Co., Ltd. (Guangzhou, China), was entrusted to determine the concentration of serum 25(OH)D_3_ with electrochemical luminescence.

### 2.3 SNP selection and genotyping

Many studies have reported that SNPs in *CYP2R1* and *CYP27B1* were significantly associated with the concentration of 25(OH)D, including rs12794714, rs1993116, rs10766197, and rs10741657 in *CYP2R1*, rs10877012, and rs4646536 in *CYP27B1(1)*. Thus, these reported SNPs were selected in this study. The SNPs associated with vitamin D deficiency in the pilot case‐control study were further investigated in family‐based study.

Single nucleotide polymorphism genotyping was completed in fluorescence quantitative PCR instrument (7500 Fast, Applied Biosystems, California, USA). All the reagents and consumables were supplied by Applied Biosystems (California, USA). All the operations were according to the manufacturer manual.

### 2.4 Statistical analysis

Categorical variable was described as frequency and percentage and compared by *chi‐square* test. Continuous variable with normal distribution was presented as means ± SD and compared with Student's *t* test. Continuous variable with skew distribution was presented as median (interquartile range) and compared with Wilcoxon rank sum test.

In order to investigate the associations of SNPs in *CYP2R1* and *CYP27B1* with vitamin D deficiency, as well as their transmission disequilibrium in vitamin D deficiency family, we analyzed the data through following strategy. On one hand, associations between SNPs and vitamin D deficiency were validated in a case‐control study by logistic regression model. On the other hand, FBAT software (V2.0.4Q, https://www.hsph.harvard.edu/fbat/fbat.htm) was applied to investigate the transmission disequilibrium of SNP in vitamin D deficiency families.^7^ The concentrations of 25(OH)D_3_ between siblings were compared with Wilcoxon rank sum test.

All the statistical analysis except FBAT was completed with SPSS 21.0 (IBM SPSS, New York, USA). Two‐tailed *P* value less than 0.05 was considered as statistical significance.

## 3 RESULTS

### 3.1 SNPs associated with vitamin D deficiency in the pilot case‐control study

The demographic and biochemical characteristics of case‐control study are shown in Table 1. The results of logistic regression model indicated that both CT and TT genotypes of rs4646536 could increase the susceptibility of vitamin D deficiency when compared with CC genotype (Table 2). The adjusted ORs were 2.846 (95%CI 1.312‐6.174, *P* = 0.008) and 2.609 (95%CI 1.197‐5.687, *P* = 0.016), respectively. No association was found for other SNPs (*P* > 0.05).

**Table 1:** Demographic and biochemical characteristics of case‐control study

Variables	25(OH)D_3_ < 20 μg/L (N = 131)	25(OH)D_3_ ≥ 20 μg/L (N = 141)	*P*
Male (%)	53 (40.5)	48 (34.0)	0.274
Age (years)	60.2 ± 11.8	58.7 ± 13.5	0.323
Smoking (%)			0.861
Never	75 (57.3)	76 (53.9)	
Ever	9 (6.9)	10 (7.1)	
Current	20 (15.3)	27 (19.1)	
Passive	27 (20.6)	28 (19.9)	
Drinking (%)			0.458
Never	119 (90.8)	122 (86.5)	
Ever	5 (3.8)	6 (4.3)	
Current	7 (5.3)	13 (9.2)	
Physical activity			0.597
Low	47 (35.9)	57 (40.4)	
Medium	29 (22.1)	25 (17.7)	
High	55 (42.0)	59 (41.8)	
BMI (kg/m^2^)	25.9 ± 3.2	25.1 ± 3.2	0.040^a^
25(OH)D_3_ (μg/L)	15.4 (14.2, 16.7)	38.9 (27.7, 75.2)	<0.001^a^

Sex, smoking, drinking, and physical activity are described as frequency and percentage and compared by chi‐square test. Age and BMI are presented as means ± SD and compared with Student's *t* test. 25(OH)D_3_ is presented as median (interquartile range) and compared with Wilcoxon rank sum test.

a Denotes the *P*‐value below 0.05.

**Table 2:** Association between SNPs and vitamin D deficiency by case‐control study

SNP	Genotype	25(OH)D_3_ < 20 μg/L (N = 131)	25(OH)D_3_ ≥ 20 μg/L (N = 141)	Adjusted OR	
				OR (95% CI)	*P*
rs12794714	GG	56 (42.7)	56 (39.7)	Reference	
	AG	48 (36.6)	66 (46.8)	0.714 (0.420‐1.212)	0.212
	AA	27 (20.6)	19 (13.5)	1.471 (0.729‐2.967)	0.281
rs1993116	CC	57 (43.5)	47 (33.3)	Reference	
	CT	56 (42.7)	71 (50.4)	0.623 (0.386‐1.104)	0.112
	TT	18 (13.7)	23 (16.3)	0.658 (0.316‐1.370)	0.263
rs10766197	GG	55 (42.0)	61 (43.3)	Reference	
	AG	50 (38.2)	63 (44.7)	0.869 (0.514‐1.468)	0.600
	AA	26 (19.8)	17 (12.1)	1.729 (0.843‐3.545)	0.135
rs10741657	GG	57 (43.5)	47 (33.3)	Reference	
	AG	56 (42.7)	71 (50.4)	0.653 (0.386‐1.104)	0.112
	AA	18 (13.7)	23 (16.3)	0.658 (0.316‐1.370)	0.263
rs10877012	GG	14 (10.7)	24 (17.0)	Reference	
	TG	61 (46.6)	61 (43.3)	1.848 (0.864‐3.949)	0.113
	TT	56 (42.7)	56 (39.7)	1.750 (0.816‐3.756)	0.151
rs4646536	CC	12 (9.2)	29 (20.6)	Reference	
	CT	63 (48.1)	58 (41.1)	2.846 (1.312‐6.174)	0.008^a^
	TT	56 (42.7)	54 (38.3)	2.609 (1.197‐5.687)	0.016^a^

Logistic regression was applied for risk assessment. BMI was adjusted to calculate the adjusted OR.

BMI, body mass index; CI, confidence interval; OR, odds ratio.

a Denotes *P*‐value below 0.05.

### 3.2 Family‐based association between rs4646536 and vitamin D deficiency

The results of family‐based association test are shown in Table 3. In the additive model, 74 informative families were included. Allele T was observed to be associated with vitamin D deficiency (*Z* = 2.248, *P* = 0.025). In recessive model, 68 informative families were included for allele T, which was associated with vitamin D deficiency (*Z* = 2.512, *P* = 0.012).

**Table 3:** Association between rs4646536 and vitamin D deficiency by family‐based study test

Model	Allele	afreq	Fam#	S‐E (S)	Var (S)	*Z*	*P*
Additive	C	0.352	74	−12.00	28.500	−2.248	0.025
	T	0.648	74	12.00	28.500	2.248	0.025^a^
Dominant	C	0.352	68	−10.75	18.313	−2.512	0.012
	T	0.648	30	1.25	6.813	0.479	0.632
Recessive	C	0.352	30	−1.25	6.813	−0.479	0.632
	T	0.648	68	10.75	18.313	2.512	0.012^a^

257 pedigrees were read in FBAT software for association analysis. S‐E (S) and Var (S) are the expected value and variance of the test statistic. *Z*: the test statistic; *P*: significance level.

The levels of serum 25(OH)D_3_ below 20 μg/L were defined as vitamin D deficiency.

afreq, frequency of allele; Fam#, number of informative families.

a The significant association between allele and vitamin D deficiency (*Z* > 0 and *P* < 0.05).

### 3.3 Association between rs4646536 and 25(OH)D_3_ between siblings

**Figure 2:**
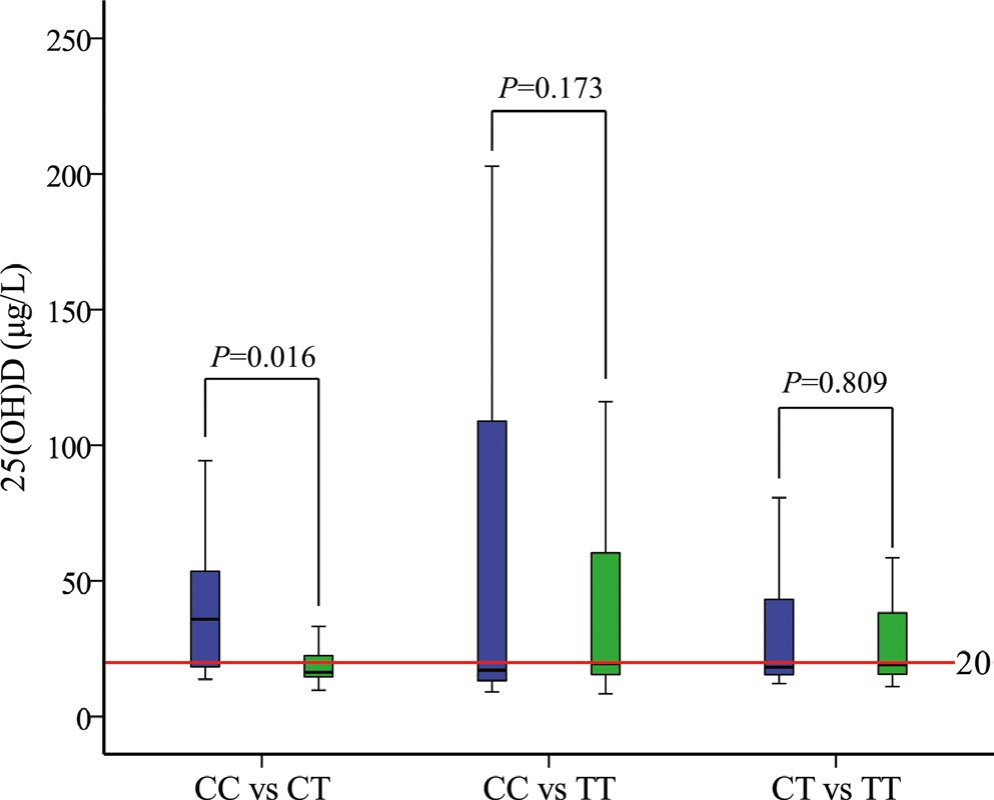
The concentration of 25(OH)D_3_ for different genotypes of rs4646536 between sibling. Fourteen pairs of siblings with genotypes of CC and CT, six pairs of siblings with genotypes of CC and TT, and 40 pairs of siblings with genotypes of CT and TT from the 257 pedigrees were included. Wilcoxon rank sum test was applied to compare the concentration of 25(OH)D_3_ between different genotypes

Fourteen pairs of siblings with genotypes of CC and CT, 6 pairs of siblings with genotypes of CC and TT, and 40 pairs of siblings with genotypes of CT and TT from the 257 pedigrees were included. The concentration of 25(OH)D_3_ for CC genotype was higher than CT genotype between siblings (*P* = 0.016, Figure 2).

## 4 DISCUSSION

We conducted three studies including case‐control study, family‐based study, and siblings study to investigate the associations between SNPs in *CYP2R1* and *CYP27B1* and vitamin D deficiency. Association between rs4646536 and vitamin D deficiency was found in case‐control study. Both genotypes TT (OR 2.609, 95%CI 1.197‐5.687, *P* = 0.016) and CT (OR 2.846, 95%CI 1.312‐6.174, *P* = 0.008) could increase a comparable risk of vitamin D deficiency, which could be explained by a dominant model of allele T that genotypes CT would have the same phenotype as genotype TT. Furthermore, family‐based associations between rs4646536 in *CYP27B1* and vitamin D deficiency were also found. There was transmission disequilibrium for allele T of rs4646536 in vitamin D deficiency families. In addition, the association between rs4646536 and 25(OH)D_3_ concentration was further verified between siblings. Therefore, these data revealed that transmission disequilibrium of risk allele T of rs4646536 contributed to vitamin D deficiency.

It was reported that the heritability of 25(OH)D ranged from 23% to 80%.^1^ The heritability of 25(OH)D was estimated to be 28.8% in the Framingham Offspring Study.^8^ Orton et al reported that the concentration of 25(OH)D had heritability up to 77%.^6^ As the key gene directly affecting the concentration of 25(OH)D, variants in *CYP27B1* may contribute to the heritability of 25(OH)D. It was verified in this study that transmission disequilibrium of allele T of rs4646536 in *CYP27B1* was associated with vitamin D deficiency.

rs4646536 is located in the 6th intron of *CYP27B1*. The function of rs4646536 is unknown. It has been reported that gene expression could be influenced by variants in intron via affecting the binding of transcription factors and splicing of mRNA.^9^, ^10^ Thus, allele variation of rs4646536 from C to T may result in abnormal expression of *CYP27B1*, which would lead to disorder of 25(OH)D concentration. It has been reported that rs4646536 was associated with the concentration of 25(OH)D as well as vitamin D‐related diseases.^6^, ^11^ Therefore, allele T of rs4646536 plus family history of vitamin D deficiency would increase the risk of vitamin D deficiency, which is significant for risk assessment, prevention and control of vitamin D deficiency.

Although association between rs4646536 and vitamin D deficiency was found in both case‐control study and family‐based study, there were also limitations in this investigation. On one hand, only six pairs of siblings with genotypes of CC and TT were included in the siblings study. The comparison result of 25(OH)D_3_ concentration for siblings with genotypes of CC and TT was inconclusive due to the small sample size. On the other hand, rs4646536 locates in *CYP27B1*, which encodes 1α‐hydroxylase catalyzing 25(OH)D to produce 1,25(OH)_2_D. The conclusion would be more convincing if the concentration of 1,25(OH)_2_D was available.

## 5 CONCLUSIONS

Allele T of rs4646536 is associated with vitamin D deficiency. Transmission disequilibrium of rs4646536 in vitamin D deficiency families contributes to the heritability of 25(OH)D.

## CONFLICT OF INTEREST

The authors declare that they have no conflict of interest.
